# Competencies required by patients and health professionals regarding telerehabilitation: A scoping review

**DOI:** 10.1177/20552076231218841

**Published:** 2023-12-11

**Authors:** Anna Lea Stark, Stephan Krayter, Christoph Dockweiler

**Affiliations:** Department Digital Health Sciences and Biomedicine, School of Life Sciences, 14312 University of Siegen, Siegen, Germany

**Keywords:** Scoping review, telerehabilitation, competence, skills, abilities, literacy, digital health, E-health

## Abstract

**Background:**

Telerehabilitation offers patients alternative access to therapy and has become more prominent during the COVID-19 pandemic. Despite the increasing attractiveness of such programs, there are research gaps regarding the required competencies in the demand-oriented technology use in rehabilitative care.

**Objective:**

The study aims at collecting evidence on competencies required by patients and health professionals for using telerehabilitation. We analyse tasks and requirements associated with telerehabilitation and derive and systematise relevant competencies.

**Methods:**

We conducted a scoping review and analysed MEDLINE, Psyndex, EMBASE, Cochrane Library, and Web of Science for empirical studies and grey literature from 2017 to May 2022. Articles had to be in English/German and refer to medical rehabilitation accompanied by health professionals taking place in the patient's home.

**Results:**

One hundred ten articles were included, covering video conferencing systems, applications with video, audio, or visual therapy content, or wearables. Depending on the program, tasks before, during, and after therapy sessions differ, as do whether these are performed by health professionals, patients, or the technology. Users need digital, health-related, social, personal, and health professionals also professional competencies. This comprises telerehabilitation, technical, health-related, and clinical knowledge, a range of physical, cognitive, social-interactive, technical, and clinical skills, a positive attitude towards telerehabilitation and experience. Whether sociodemographic factors promote successful use is unclear.

**Conclusions:**

Telerehabilitation requires a variety of different competencies from patients and health professionals — going beyond the sphere of technical skills. This highlights the need for an evaluation of existing programs for promoting competencies in the use of telerehabilitation and refinement of the programs in line with demands.

## Introduction

### Telerehabilitation

The COVID-19 pandemic greatly impacted those in need of medical or rehabilitative services. To stop transmissions of the disease, facilities had to reduce or shut down their centre-based rehabilitation programs.^1–3^ In this context, telerehabilitation was an alternative for patients not willing or able to use analogue services.

Telerehabilitation is defined as the delivery of medical rehabilitation via information and communication technologies (ICTs).^4,5^ The possible fields of application are heterogeneous and differ depending on the indication, therapy form, and health professionals involved.^6^ Various technologies and tools, such as sensors, video conferencing systems, virtual reality, or messaging systems, can be used either synchronously or asynchronously.^6,7^ Recent reviews show that (e.g. physical or cardiac) telerehabilitation is effective and comparable to in-person rehabilitation.^8,9^

### Competencies for using digital health services

The use of telerehabilitation places various demands on users, and different competencies are required for successful application. Competence in this context means ‘a measurable human capability that is required for effective performance […] [that] may be comprised of knowledge, a single skill or ability, a personal characteristic, or a cluster of two or more of these attributes’.^10^ In the context of telerehabilitation, experiences during the pandemic show that inadequate training of health professionals is a barrier to successful implementation and usage.^1^ Further, studies identified that a lack of digital and health-related competencies on the part of patients and health professionals can hinder successful services.^11–13^

Research on competencies regarding digital health services is already taking place in the fields of telemedicine, telehealth, and eHealth. Interestingly, competence analyses in these fields so far only examined health professionals as a target group.^14–17^ Regarding telehealth, competence models have been developed for various professions (e.g. non-physician telemedicine employees or telehealth nurses). These differ in their complexity and differentiate various competence domains or levels.^10,16,17^ With such a competence model – defined as ‘an organising framework that lists the competencies’^10^ – it is possible to outline the characteristics of a competent group of persons. It has been shown that orientation towards methodological guidelines, e.g. the manual by Marrelli et al.,^10^ can strengthen the validity and utility of such competence models.^18^ However, the developed models are not yet based on theories or adherent to specific guidelines.^14–18^

### The eHealth literacy model

Related to the competent use of digital health services is the concept of eHealth Literacy, defined by Norman and Skinner^19^ as ‘the ability to seek, find, understand, and appraise health information from electronic sources and apply the knowledge gained to addressing or solving a health problem’. Their eHealth literacy model differentiates traditional literacy (ability to read/speak/write), computer literacy (ability to use computers to solve problems), health literacy (skills to interact with health systems/engage in self-care), as well as information, media, and science literacy (skills in selecting, evaluating, and using media/research/information).

So far, there is little research on eHealth literacy in the context of telerehabilitation. A recent review on the application of eHealth literacy in digital health interventions found that only two of 158 records focus on telerehabilitation.^20^ Often, studies examine the effect of a telerehabilitation program on eHealth literacy rather than whether the success of a program depends on the eHealth literacy level.^21^

### Research gaps and study aims

As shown before, research on telehealth competencies has already progressed, but it remains unclear which competencies are required for using telerehabilitation. Further, it lacks research on the part of patients’ competencies and guideline-oriented competence frameworks. We aim to fill this research gap by collating and mapping evidence on telerehabilitation competencies. By doing so, the review is a first step to develop a competence framework for telerehabilitation. Following Marrelli et al.,^10^ a review serves to develop a preliminary list of competencies and supplements other data collection methods for competence modelling. In addition, the results of this review can also be used to develop needs-oriented training for telerehabilitation users and thus eliminate competence deficits in the future. Therefore, the following primary research question will be answered: What evidence exists about the competencies of patients and health professionals required for the use of telerehabilitation? We will address the following subquestions: (1) Which competencies necessary for using telerehabilitation have been identified until now? (2) What tasks and requirements are associated with the use of telerehabilitation and what competencies can be derived from this? (3) What research gaps can be identified?

## Methods

In line with our objectives, we conducted a scoping review following Levac et al.,^22^ as this is a suitable method to examine the range of literature, systematise it and identify research gaps. The six steps are described below. We follow the Preferred Reporting Items for Systematic Reviews and Meta-Analyses extension for Scoping Reviews (PRISMA-ScR) checklist (see Appendix 1).^23^ The review protocol is available at https://osf.io/b6dzp.

### Identifying the research questions

Two reviewers (ALS and SK) developed the research questions through an iterative process including exploratory literature screenings, an exploration of relevant terms/concepts, and thematic discussions.

### Identifying relevant studies

We developed the search strategy with the support of the University of Siegen library and another expert in telerehabilitation, ensuring content and methodological expertise. The iterative process included an initial search in MEDLINE and Web of Science, analysing keywords and indexed words used for the topic, and reviewing articles for inclusion. For the final search, we included five databases: MEDLINE, Psyndex, EMBASE, Cochrane Library, and Web of Science. Search terms refer to the categories rehabilitation, technologies, and competencies, including Medical Subject Headings (MeSH terms) and synonyms. As telerehabilitation is seen as a rapidly developing field,^24^ and in line with the reviewers’ spoken languages, we restricted the search by database filters to articles in English or German from 2017 to 2022 (date of search: 10/05/22). We restricted the search to the presence of search terms in the title/abstract, as both provide substantial information and focus of the article.^25^ The search strategy for MEDLINE is shown in Appendix 2.

### Study selection

We defined coding instructions for the title/abstract and full-text screening using the Population, Concept, and Context principle.^26^ These are summarised in the following Table 1.

**Table 1.** Inclusion and exclusion criteria using the PCC principle.

PCC	Inclusion criteria	Exclusion criteria
Population	Patients defined as people with a disease (regardless of indication) using telerehabilitation Health professionals providing telerehabilitation, incl. therapists, physicians, psychologists, or nurses^27^	Persons not belonging to the direct user group, such as managers of rehabilitation facilities
Concept	Telerehabilitation defined as medical rehabilitation via ICTs accompanied by health professionals in a one-to-one or group format Medical rehabilitation includes treatment for recovering from damages or diseases provided by different specialists (e.g. physicians, psychologists, or therapists) from different disciplines^27,28^ Competencies (including knowledge, skills, attitudes, and personal characteristics) or tasks/requirements from which competencies can be derived	Programs without the company of health professionals (e.g. pure online information, standalone monitoring systems or commercial health apps/gadgets) Programs for job-related rehabilitation or reintegration
Context	The telerehabilitation program is used by patients in their home environment	Rehabilitation taking place at a supervised or inpatient environment, or at patients’ workplace
Formal criteria	Articles in English or German from 01/2017 to 05/2022 Empirical research (all study designs), reviews, grey literature (e.g. technology description, discussion paper)	Abstracts, posters, anthologies, protocols, no full-text access

PCC: Population, Concept, and Context.

The exclusion of supervised or inpatient settings was necessary because the use of telerehabilitation in hospitals, care facilities or the workplace is associated with different underlying conditions and requirements for users compared to use at home. For example, the spatial conditions, the equipment and the possibilities for technical or social support in the use of technology differ. A different requirements and skills profile is likely depending on the setting.

Two reviewers (ALS and SK) test-coded a randomised sample of 614 articles (confidence level of 99%, population of 8165 articles)^29^ to check the fit of the coding instructions and to calculate intercoder reliability using Cohens Kappa. Values for Cohens Kappa range from 0 to 1, whereby values ≥ 0.61 are seen as a substantial level of agreement.^30^ Test coding revealed κ = 0.672 and accordingly, our coding instructions could be approved for further analysis. Afterwards, we distributed the remaining articles halfway to both reviewers for final coding. Due to the large number of articles both reviewers shared the inclusion/exclusion process using the software Rayyan, starting with screening titles/abstracts in the first round and full texts in the second round. Uncertainties were discussed between the reviewers.

### Charting the data

We developed a data-charting form, which we piloted and modified continuously. We extracted descriptive metadata of the articles, namely first author, year of publication, country of research, study design, and primary study outcome. Further, we captured outcomes relevant to answering the research questions, namely technology type or indication group. The user group and competencies were charted separately building on inductive and deductive content analysis using the software MAXQDA. The coding scheme that was developed to describe and systematise the required competencies is presented in the next section. The data was charted by one researcher and checked by another.

### Collating, summarising, and reporting the results

We first analysed the results, including numerical summary analyses and structuring content analysis (following Kuckartz^31^). The coding scheme was formed deductively based on the competence components and inductively expanded in the process. According to the process of Marrelli et al.,^10^ we thereby identified required knowledge, skills/abilities, attitudes, and personal characteristics, and inductively added experiences. We combined similar characteristics to a category, labelled and defined these competencies, and lastly created an initial list of competencies. In the results section, we will answer the research questions and present the descriptive summaries and content analysis alongside the main coding categories. The discussion section will focus on implications for research, practice, and policy. We did not evaluate the methodological quality of the articles because of the variations in their contents (e.g. study design, target group, intervention, and goals).

### Consultation

A librarian and an expert on telerehabilitation supported the review process through consultations to discuss and optimise the methodology. To transfer the results and for transparency, we published a study protocol and we will disseminate the results in the science and practice community (e.g. on scientific conferences, within the final report or via presentations for interested patients and rehabilitation facilities).

## Results

In the following, we first describe the process of study selection and the characteristics of included studies (publication year, country, study type, and objective). It follows a description of telerehabilitation programs, including the focused indication and technology used. Then, the results of the content analysis are presented to answer the research questions. First, the tasks associated with the use of telerehabilitation are described. Second, the competencies required for telerehabilitation are presented. Content analysis revealed the following central categories: required literacies, knowledge, skills, attitudes, personal characteristics, and experience.

### Article selection

**Figure 1.**
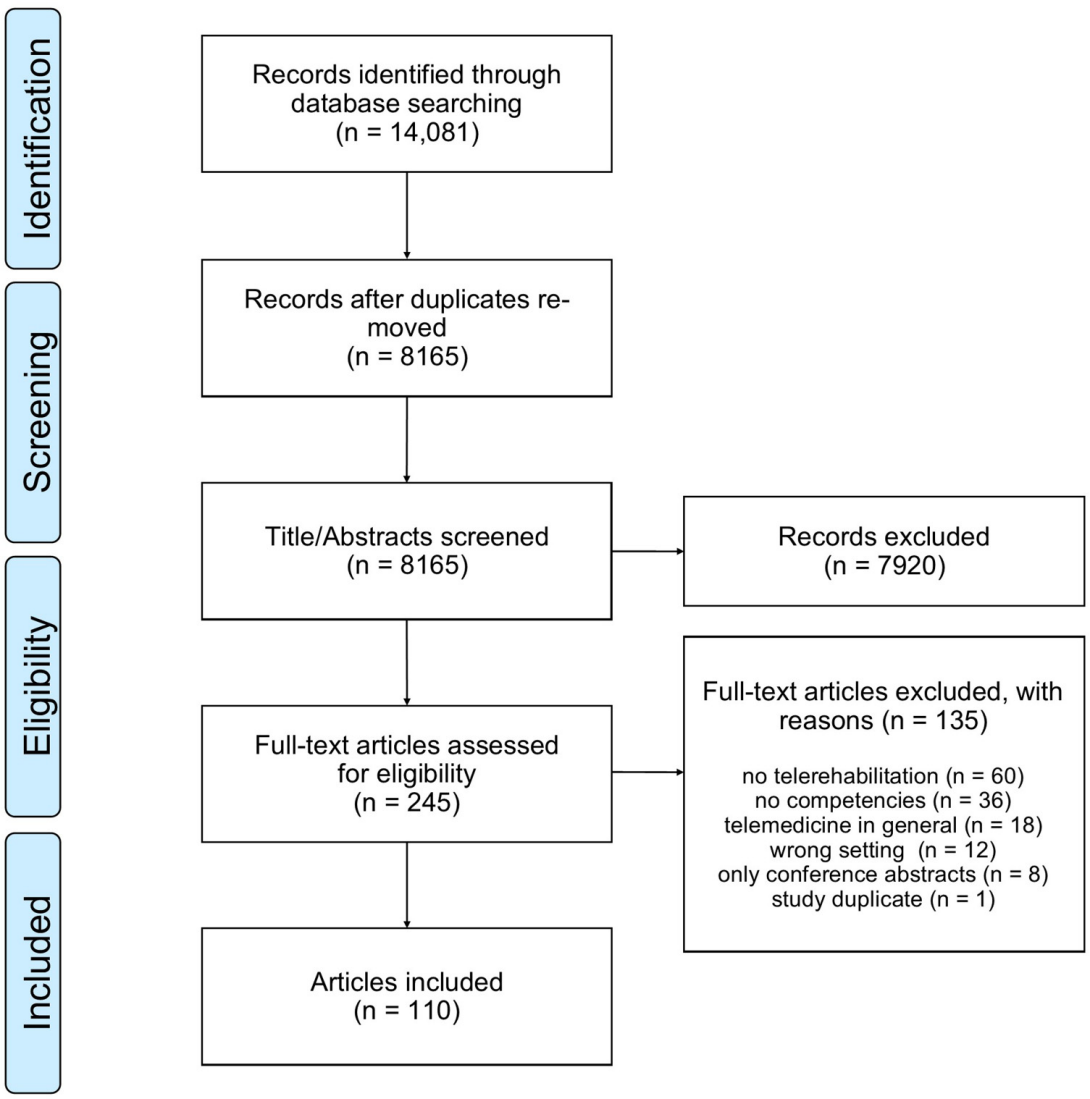
PRISMA flowchart.

The search yielded in 14,081 articles, of which 5916 were duplicates. Of the remaining 8165 articles, titles/abstracts were screened. Two hundred forty-five articles were eligible for full-text screening. From these, 135 articles were excluded because they were conference abstracts, did not meet our definition of telerehabilitation, did not focus on the home setting or competencies, dealt with telehealth in general or were a study duplicate. Hence, 110 articles were included. The PRISMA flowchart^32^ is shown in Figure 1. The data extraction chart is provided in Appendix 3.

PRISMA: Preferred Reporting Items for Systematic Reviews and Meta-Analyses.

### Study characteristics

***Year and Country.*** The included articles are published from 2017 to May 2022, with the fewest (*n* = 12) in 2017 and the most (*n* = 33) in 2021. In 39.1% (*n* = 43) research is conducted in Europe, with the largest share in Great Britain (*n* = 8), 22.7% (*n* = 25) come from North America, mostly the United States (*n* = 17), 17.3% (*n* = 19) of research is conducted in Asia and 15.5% (*n* = 17) in Australia/New Zealand. Only few are from Africa (*n* = 3; 2.7%) or South America (*n* = 1; 0.9%) or research is conducted cross-continental (*n* = 2; 1.8%).

***Study Design.*** One hundred four (94.5%) articles are empirical studies and six (5.5%) theoretical articles (authors’ opinion, technology descriptions). The following study designs occur: 61 (55.5%) cross-sectional, 13 (11.8%) reviews (literature reviews, project overviews), 12 (10.9%) pre-test/post-test without a control group, nine (8.2%) randomised controlled trials, four (3.6%) case studies, three (2.7%) longitudinal studies, and two (1.8%) controlled trials without randomisation. Of 104 empirical studies, 43 are quantitative (41.3%), 26 (25.0%) mixed-methods, 22 (21.2%) qualitative, and 13 (12.5%) secondary data analyses.

***Research objective**.* Most commonly the articles examine the experience of telerehabilitation users (24.5%), implementation factors (16.4%), feasibility of a program (15.5%) or perceptions regarding telerehabilitation (15.5%). 11.8% analyse acceptance of and 10.5% attitudes towards telerehabilitation. Further 10.5% examine the usability of a program.

### Characteristics of telerehabilitation programs

**Figure 2.**
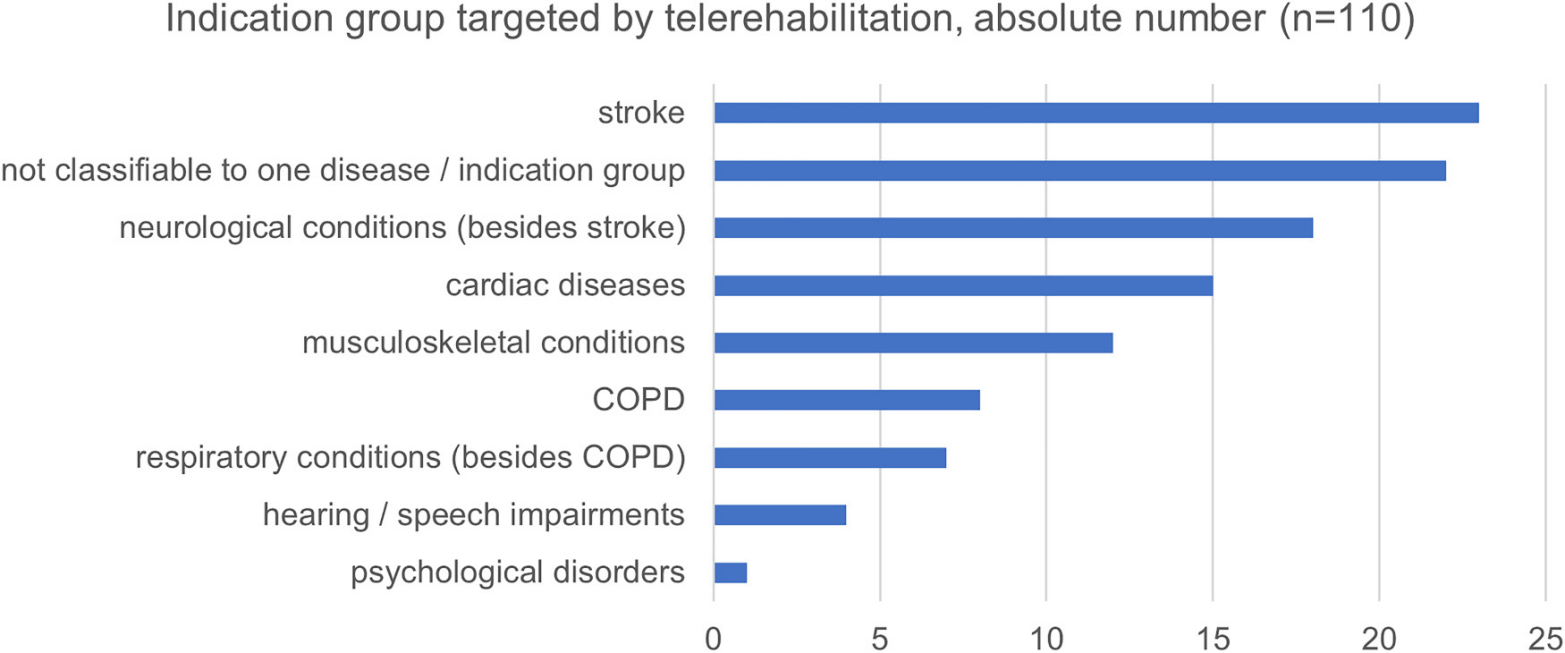
Indication group targeted by telerehabilitation programs of included articles (*n* = 110).

***Focused indication***. Programs focus on different diseases, mostly stroke (20.9%), other neurological conditions (16.4%), or cardiac diseases (13.6%). See Figure 2 for all indications.

***Technology type.*** The largest proportion (33.6%) reports on various technologies^33^ or telerehabilitation in general.^34^ 21.8% present a videoconferencing system (e.g. Skype) in group sessions^35^ or one-to-one,^36^ used directly for therapy delivery (e.g. physical/speech training),^37^ monitoring or education.^36,38^ In 17.3%, programs comprise smartphone, tablet or computer apps with video, audio or text-based health/training content.^39^ Apps include goal setting, behaviour tracking or digital games.^40^ Further features are automatic reminders, feedback or messaging with a health professional.^39,41–44^ In 14.5%, apps are combined with wearables for tracking the health status, emergency situations, or training progress.^45^ Sensors are usually connected with a smartphone and allow automated data uploads or alarms.^46^ Further 6.4% are gaming devices. Consoles (e.g. Xbox) and gaming equipment (e.g. balance boards) are applied for therapeutic games,^47–50^ accompanied by health professionals, e.g. via video calls.^49^ 2.6% of articles report on robotic systems, e.g. exoskeletons for movement training.^51–53^ Lastly, 3.6% of programs are classified as ‘others’ and comprise e.g. custom-designed tools.^54^ Eight articles include Artificial Intelligence, Virtual or Augmented Reality.^55^ Additionally, health professionals use digital interfaces to communicate with or monitor their patients.^45,46,56^

### Tasks associated with the use of telerehabilitation

***Tasks before starting a telerehabilitation session.*** Twenty-seven articles describe health professional training carried out by technology vendors or experienced health professionals in-person or remotely.^57,58^ It teaches how to use the technology and execute the therapy, including benefits/risks, legal/ethical considerations, troubleshooting technology issues,^37,54,59–61^ educating patients or conducting quality improvements.^62^ Twenty-two articles describe patient training for using the technology and putting on devices,^63–65^ conducted by researchers or health professionals analogue or via videoconference.^59,66,67^ Several articles recommend hands-on-training and trouble-shooting sessions for both user groups.^68,69^ Partly, patients are provided with written/oral instructions for technology usage/set up,^42,70^ including exercise rules,^71^ emergency contact information,^38^ or online behavioural etiquettes and privacy rules.^62^ Health professionals are partly provided with written material about digital therapy.^55^ Introducing patients to the technology takes place via meetings at a rehabilitative facility,^46,72,73^ during inpatient rehabilitation,^74^ at home or online, e.g. during the first online therapy session.^55^ It is conducted by health professionals or study staff^41,59,75^ to get in touch with the technology, ensure safe usage, and identify support needs.^50,51,76^ Technology introduction to health professionals is not mentioned. A next step is the provision with and set up of hardware/software at the patients’ home, done by health professionals/technicians via house visits, or by patients/caregivers.^66,71,77–80^ Technology set-up at the health professionals’ workplace is not described. Partly, health professionals can tailor the program/therapy content to patients’ needs before it starts,^47,57,81^ sometimes together with patients/caregivers.^81^ The last step is the practical preparation before each therapy session. For patients, this includes turning the device on, preparing equipment, and putting devices on.^62,82,83^ These steps are not described for health professionals, but logically, for synchronous sessions, apply to them too.

***Tasks during a telerehabilitation session.*** First, the patient needs to execute the training, in synchronous sessions together with the health professional. According to the technology type, this comprises doing videocalls, exercising with an app or reading/watching educational material. Patients usually receive instructions on how to perform exercises, which are displayed in the app^84^ or given by the health professional during therapy.^74,82^ Another step is the real-time tailoring and adaption of therapy^85^ to patient's needs and progress: ‘This can be done either automatically by means of a decision algorithm or manually with the help of a therapist.’^86^ Some programs include different difficulty levels for patients to choose from.^87^ During digital therapy, there may occur medical issues or emergencies, that the patient or health professional needs to handle.^84^ Party, the digital device comprises channels to communicate with the health professional.^43,62,84^ Further, the patient exercising independently, needs to be able to self-monitor and to know symptoms that present an emergency.^62^ Additionally, the technology can detect an emergency and take steps automatically (e.g. alert someone).^84,88^ A majority of articles report that technical problems (poor internet connections,^60,74,89,90^ problems with audio/video quality,^37^ or software breakdowns^71^) occurred. Partly, patients solve these problems,^82^ partly caregivers, health professionals or IT departments give support.^68,91,92^ Furthermore, programs can comprise synchronous monitoring of patients and provision of real-time feedback and motivation.^93^ During sessions, health professionals can provide feedback, encouragement, motivation,^94^ supervision, or safeguard.^47^ In one study an avatar gave real-time feedback.^66,95^ Further, technologies can ‘enable remote monitoring of physiological signs and symptoms during exercise in real-time.’^96^

***Tasks after a telerehabilitation session.*** After a session, follow-up needs to be done. This includes putting down wearables, switching off devices, and charging/storing them.^60,68,82,97^ Partly, health professionals document the session and record patient/therapy data.^47^ Record-taking can also be automated by wearables/apps.^42,98,99^ Adaptation of the program to the patient's needs and progress can take place after a session by the health professional, asynchronously or in real-time with the patient.^100^ Also, monitoring of patient data and providing feedback/motivation can take place asynchronously after a session through health professionals or technology.^101^ Some articles address self-motivation as a patient or caregiver task.^49,67^ Partly, the app contains motivational elements, e.g. goal-setting or gamification.^43,88,102^ Another task is reminding patients of exercises/appointments. This is done by the health professional,^34^ apps (e.g. automated pop-ups),^41^ or patient (e.g. calendar notes).^68^ Partly, there is a final visit to the patients’ homes after programme completion for data measurement or removal of devices.^49,66^

***Changing tasks and roles.*** It is noticeable that one telerehabilitation task can either be performed by the patient, health professional or technology, varying by program. On one hand, telerehabilitation adds new tasks (e.g. technology set-up) for health professionals.^63^ On the other hand, some tasks performed in analogue rehabilitation can be taken over by patients or technology.^47,63,103^ The emergence of new work tasks and the elimination of traditional tasks can change the role of health professionals and redistribute responsibilities.^103,104^ Thus, telerehabilitation ‘is a different way of providing care that redefines the health professionals’ identity’.^63^ Further, it leads to new roles and professions. Some studies refer to a new coaching role, going hand in hand with the supervision/motivation required for telerehabilitation.^105^ Partly, health professionals work as telehealth managers or telehealth professionals in telehealth clinics.^33,79^ In some cases health professionals do not see the mentioned tasks as part of their professional role (e.g. fixing technical issues).^63^ Instead, technicians should be hired for this purpose.^63,97^ Also for patients, it shows up that with telerehabilitation new tasks must be taken over.^46^ As a result, the role image changes as telerehabilitation alters ‘understandings about […] what it means to be a patient experiencing rehabilitation remotely’.^79^

### Required competencies

The required competencies vary according to technology type, program design, and user group. In the following, we examine the required literacies in general and then the competence components of knowledge, skills, attitudes, personal characteristics, and experience.

***Required literacies.*** In general, articles mention a high level of health literacy, digital literacy (or technology, computer, mobile phone, or e-literacy), and eHealth literacy (or digital health or telehealth literacy) as necessary for both user groups.^46,106–111^

***Required knowledge.*** Program usage requires health professionals and patients to have knowledge about telerehabilitation. First, users need to be aware of available programs and technologies^112–117^ and need in-depth knowledge.^42,118^ For health professionals, this includes telerehabilitation components,^115^ benefits,^119^ policies/guidelines,^98^ impacts on workflows, workload, and responsibilities,^107,120^ implementation strategies,^104^ and impacts on patients.^107^ Regarding patients, knowledge about telerehabilitation components, methods, risks, and benefits are mentioned.^42,62,121,122^ In view of the above-mentioned tasks, health professionals need to be aware of legal/ethical considerations, techniques/processes, risks, and how to do patient training, demonstrations, monitoring, guidance, feedback, or documentation remotely. Both groups should know behavioural etiquettes for remote communication.

Further, a lack of technology-related knowledge is a barrier to successfully using telerehabilitation. Articles identify a lack of technical knowledge for patients and health professionals,^59,80,118,123^ and of knowledge on how to use a device for patients.^107,124,125^ Further, data security knowledge for both user groups,^108^ and knowledge of privacy regulation for health professionals is required.^60,104^ In view of the above-mentioned tasks, users need to know how to set up hardware/software, don wearables, adapt a program, or handle technical issues.

Health-related knowledge is addressed sporadically. One study states that ‘a lack of knowledge of rehabilitation and how it can occur may be a barrier to engagement with the deployed technology’.^126^ The need for health knowledge is mentioned for programs with a high degree of self-management. Accordingly, patients need ‘to be aware of their current body condition and changes in their body’.^112^ Knowledge of diseases and exercises is required to ‘know what physiological reactions are acceptable to experience during remote exercise […] [and] what to expect while exercising, especially in relation to any symptoms that may suggest an adverse event’.^88^ Both user groups need to know what to do in a medical emergency.^94^

Lastly, clinical knowledge related to the job of health professionals is mentioned. On one hand, knowledge obtained during professional training is necessary, on the other hand, practical experience gained through working as a health professional.^60,120,127^ Further, health professionals need to know how to assure and improve the quality of digital therapies.^62,103^

Further, the technology itself can be provided with knowledge. This can comprise ‘a combination of medical, psychological, and technical knowledge in the form of embedded algorithms analysing and processing online the data generated by digital technologies. As such, clinical artificial intelligence is expected to play a key role in clinical decision making, online adaptation of therapy exercises, and monitoring of progress […].’^51^

***Required skills*.** Telerehabilitation requires physical, cognitive, social-interactive, technical, and clinical skills. Most required physical skills are disease-related. Thus, patients with specific/severe diseases are excluded from the program or have difficulties using it.^88,101^ In order to execute physical exercises, physical functionality must be present.^68,77,128^ Studies show that impaired fine motor skills can restrict the use of ICTs.^68,129^ Also, patients’ participation may be unsafe due to a poor mobility that constitutes a risk of falling while performing exercises remotely.^75,80^ Additionally, the general fitness level is mentioned as a requirement,^51,88^ and age-related impairments as barriers. Thus, ‘aging is […] accompanied by important changes in visual acuity and manual dexterity, which limits the potential use of small-screen devices’.^130^ Articles also refer to hearing deficiencies as barriers.^74,77,115^ Contrary, Pitt et al.^37^ conclude that patients with severe language impairments and advanced age are able to use their program effectively. Other articles declare that their programs are developed in line with the users’ physical skills.^39,50,87,131^ All mentioned physical skills refer to patients.

Regarding the cognitive skills of patients, cognitive impairments are mentioned as barriers or exclusion reasons.^34^ In the context of aging, older patients may have difficulties processing complex information, comprehending or learning new skills as required for telerehabilitation.^75,132^ The required cognitive skills depend on the technology, e.g. the therapy duration, complexity of instructions, and cognitive requirements differ.^50^ E.g., in a videoconferencing program patients need a higher concentration compared to analogue therapy.^57^ Contrary, some articles conclude, that patients with cognitive impairments are able to use their technology effectively.^71,133^ Furthermore, reading, spelling, and writing skills are necessary.^40,109^ Additionally, coping and reflection skills are mentioned as facilitators.^35,134^ Another cognitive skill is self-management, as some programs require more personal contribution.^46,104^ Related to this is the ability to motivate oneself and self-discipline.^46,67,75^

Regarding cognitive skills of health professionals, it is stated that with telerehabilitation more independence and self-management is needed.^55,60^ Next, creativity is needed ‘to balance out some features missing in telerehabilitation.’^80^ Due to the rapid and partly unstructured implementation of programs during the pandemic creative solutions were required from professionals, e.g. when not enough equipment was available.^60,61^ Following this, health professionals also need adaptability and flexibility regarding digital therapy, which partly needs to be adapted to the patients’ needs. Adaptivity is also needed in order ‘to modify […] materials, their educational techniques, and the setting’^81^ as required.^57^ Adapting to online therapy and new environment concerns health professionals and patients.^37^ Further, health professionals need the skill to critically reflect technology usage and anticipate challenges of patients.^37,50,103^ In the context of the telerehabilitation tasks, both user groups need to be self-aware and aware of their surroundings during therapy to comply with the behavioural etiquette. Further, to manage technical difficulties they need to have problem-solving skills.

The technical skills of patients comprise skills in accessing and setting up technology, donning wearables, downloading/navigating a program, communicating via digital devices, adjusting program settings, or maintaining the technology.^50,80,82,88,101,115,135–137^ Technical skills of health professionals concern technology set up and usage.^60,88^ However, technical skills are often not further specified.^49,74,103,137^ Contrary, in Fang et al.^93^ and Dodakian et al.^99^ it is shown that patients with limited technical skills are able to use the programs effectively.

Next, we will consider required social-interactive skills. First, health professionals must have the ‘ability to create and sustain good human connections with patients’ and between patients.^104,106^ Thus, they require the skill to initiate social interaction, and to be emphatic and active listeners.^94,106^ Next, health professionals need skills to motivate patients, despite their physical absence.^67,71^ Further communication skills include ‘creating a supportive communication environment […], facilitating communication between the group members and managing group dynamics in the online environment’.^37^ Users need skills to communicate remotely, e.g. not to interrupt each other.^62,63^ In difference to analogue therapy they need ‘heightened levels of expression to compensate for limitations in other aspects of non-verbal communication, such as body language, which may not be seen on camera’.^119^

A job-related skill of health professionals is the remote monitoring and analysing of patients’ data, clinical status, safety, and progress via digital devices.^100,104,131^ Referring to the mentioned coaching role, health coaching (going hand in hand with motivation skills) is a new required skill.^103^ Further, skills in remote diagnose, e.g. of muscle problems during physiotherapy, are needed.^122^ In a program with digital games, the following clinical skills are required: ‘selecting appropriate systems, clients, and games, grading activities, evaluating outcomes, and integrating theoretical approaches […] to gaming-based treatment’.^50^ Regarding telerehabilitation tasks, health professionals need the skill to remotely guide patients through therapy, give feedback, document data, and perform quality management.

***Required attitudes*.** In general, a positive attitude towards telerehabilitation is a requirement for successful technology uptake and usage.^37,52,63,115,138^ First, being interested in technology or telerehabilitation and being willing to learn is a prerequisite,^57,116,120,129^ whereas lack of interest was a reason to decline/drop-out of programs for patients.^58,71^ Second, being motivated is a beneficial attitude, as it promotes adherence to digital therapy.^57,60,67,127^ Being committed to and engaged with the program is a requirement for health professionals.^76,103^ Additionally, being enthusiastic about the program as a health professional ‘is likely to be a major contributor to the high adherence rates’^92^ of patients. Commitment, engagement, and enthusiasm are also discussed from the patients’ side as facilitators.^91,122,134,139^ Further, health professionals need to be open-minded about the patients’ ability to use technology,^60^ and patients need openness towards telehealth.^36^ A further contributor to patients’ adherence is that the health professional is the patient.^92^ Next, patients’ sense of competence is discussed as a hindrance, if they think that they are unable to use the device.^101^ In this context, users’ belief in their capabilities (self-efficacy) is beneficial.^63,67^ Partly, high technology affinity is considered important.^57,127^ Thus, difficulties with technology usage on the patients’ side represent inadequate coping with technology and hinder effective usage.^42,67,68^ Lastly, acceptance is discussed as a facilitator for program uptake, adherence, and effective usage.^97,132^ Some articles indicate that both user groups accept telerehabilitation,^76,100,112,139^ whereas others point out lacking acceptance.^69,98^

A negative attitude, including scepticism, concerns, and fear towards telerehabilitation, on the other hand, hinders technology uptake/usage.^115^ Concerns of patients refer to trust, security, privacy,^112,134^ impact,^134^ effectiveness,^122^ their competence, confidence and comfort in usage,^67,109^ or lack of social interactions.^91^ Health professionals’ concerns involve changes in their work role, tasks, or responsibilities,^98,103^ relationships with patients,^129^ costs,^60^ technical issues,^89^ users’ competencies/confidence,^107,109^ data privacy and other ethical/legal concerns,^51,108,117^ program quality/reliability,^116,140^ or user-friendliness.^106^

***Required personal characteristics*.** Sociodemographic factors can affect the usage of telerehabilitation. Considering the age, discrepancies become apparent. While in some studies patients’ age has an impact on successful use (older age as inhibiting),^122,129^ others exclude such an effect.^78,132^ Morris et al.^116^ conclude that the age of health professionals can impact telerehabilitation implementation. Regarding the gender of patients, some studies declare that the effectiveness of telerehabilitation differs between men and women (female as beneficial),^75,112,127^ whereas others do not identify differences.^78,115^ A lower level of education can be a barrier for patients to use telerehabilitation.^122,124,129^ Other studies see telerehabilitation as an efficient option for patients with lower education,^93^ or do not see an effect.^115^ Further, being employed and previous employment can influence patients’ program uptake.^43,130^ Regarding health professionals, a longer time in the profession can impact the implementation.^116^ Being a ‘hands-on’ therapist is partly seen as a hindrance.^115,119,137^ Dahmen et al.^127^ present the socioeconomic status as a possible reason for effective program use by younger patients, and Ghaldiri et al.^110^ describe socioeconomic disparities as barriers to program uptake. The place of residence/housing situation of patients is important for program uptake/usage in some studies,^78,79,103^ in others not.^66,132,139^ Further, the cultural origin can affect program adoption/uptake, but research needs are announced.^98,132^

Further, disease characteristics can influence the use of telerehabilitation. For example the general health status can affect learning and using telerehabilitation.^129^ In Munsell et al.^78^ the disease phase impacts engagement with digital therapy, whereas disease duration has no influence in another study.^132^ Severe comorbidities, intense symptoms, and complex diseases can lead to a higher demand for support in using the program.^70,103^ Next, a ‘difficult’ personality of patients is discussed as a barrier. In a program with videoconferencing,^37^ health professionals stated that some dominant personalities are difficult to manage remotely. Last, user's confidence in general and in using technology is a required personality trait.^68,89,98,107,109,122^

***Required experience*.** Experience with technologies or telerehabilitation is seen as beneficial for effective program uptake or usage,^41,58,124^ whereas lacking experience is seen as a hindrance.^63,74,96,98^ In contrast, some articles conclude ‘that previous experience with technology did not seem to be a major barrier for technology use’^68^ and that even with prior technology experience, telerehabilitation was often ‘too big a jump’ for patients.^141^

**Figure 3.**
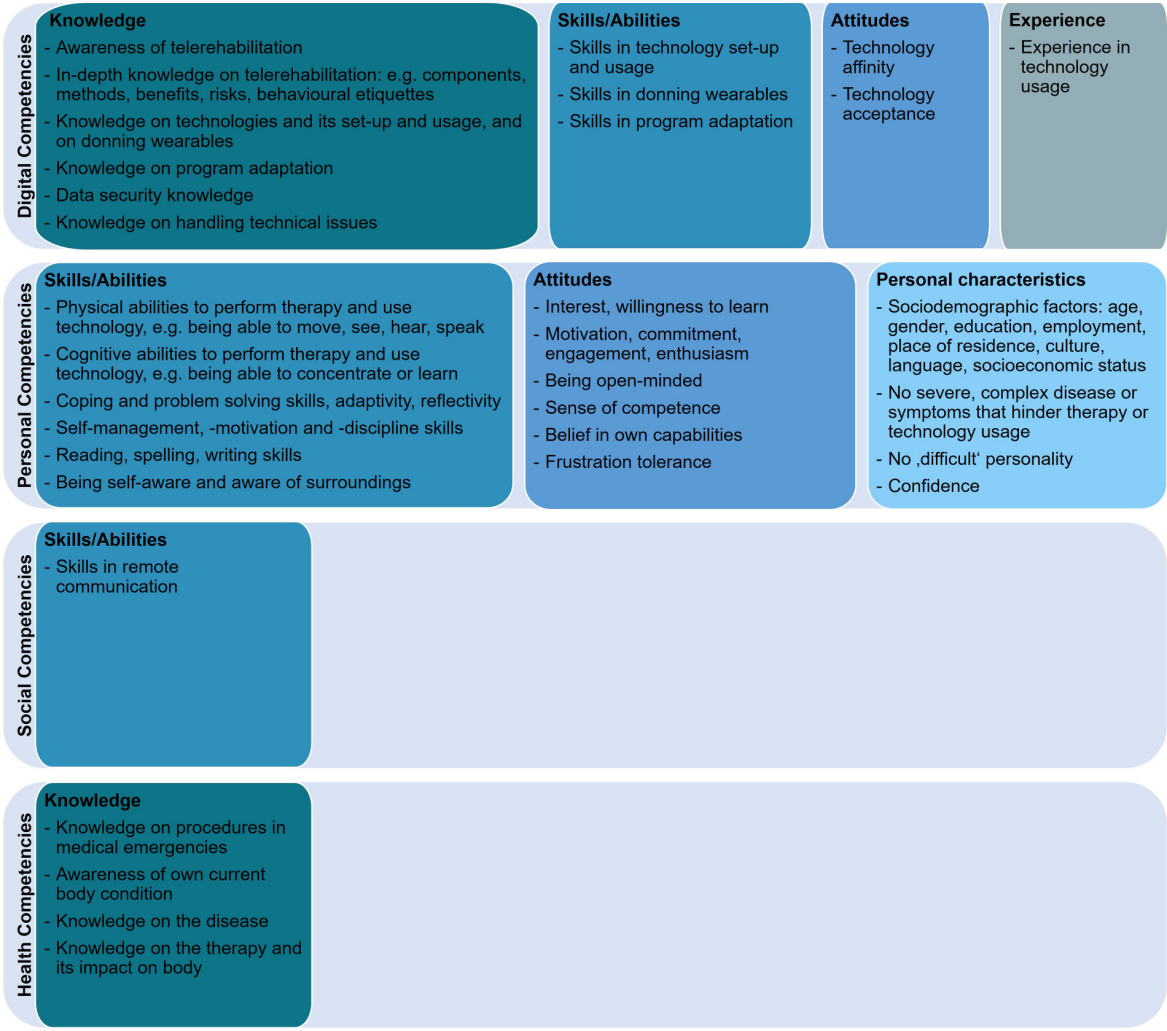
Preliminary overview of telerehabilitation competencies required by patients.

**Figure 4.**
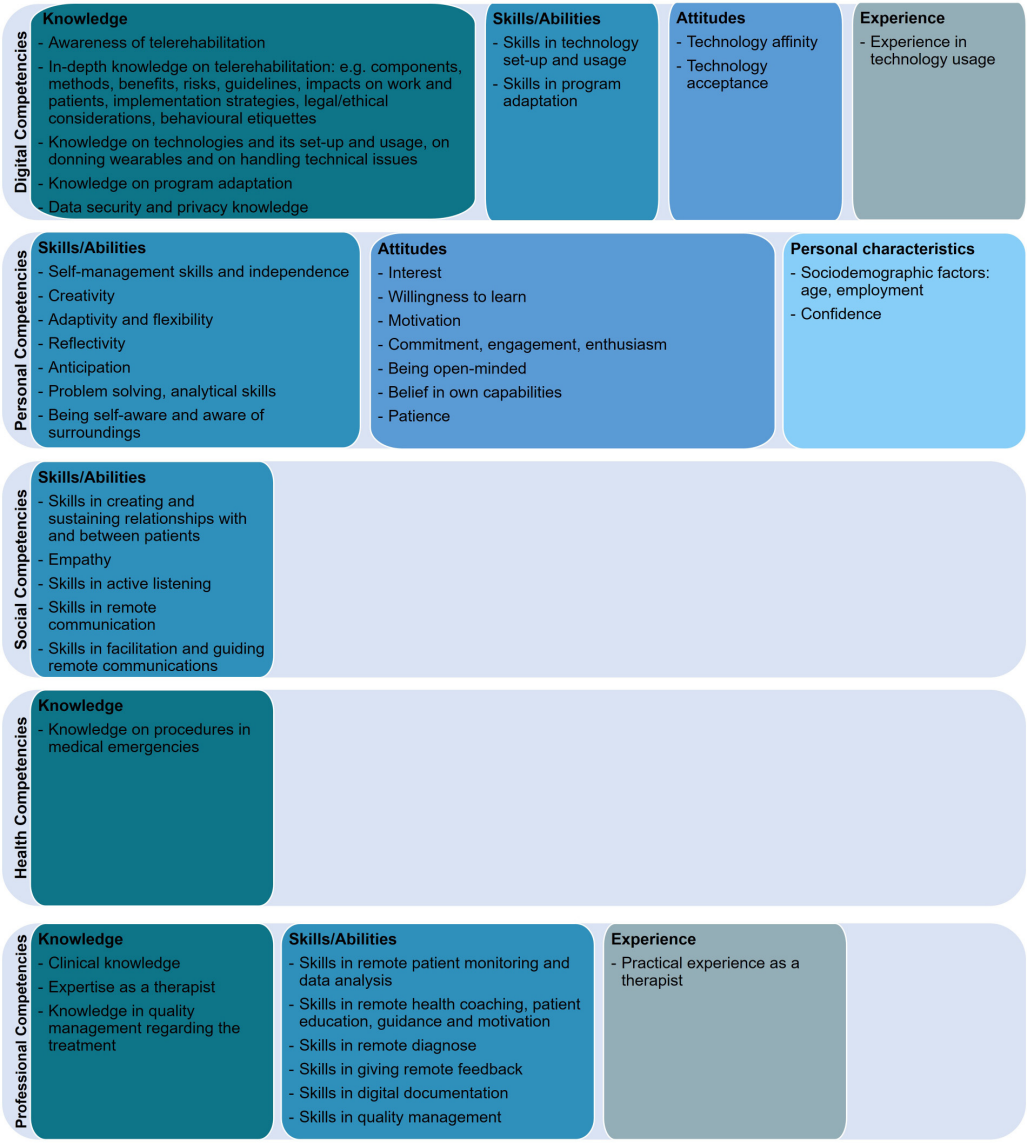
Preliminary overview of telerehabilitation competencies required by health professionals.

In Figures 3 and 4 results are summarised by patients and health professionals.

## Discussion

### Summary of evidence

The scoping review aimed at mapping evidence on competencies in telerehabilitation. We included 110 articles on video conferencing systems, programs with video, audio, or visual therapy content, and wearables. Scientific articles consider competencies only peripherally when analysing implementation factors, usability, or acceptability of such programs. We found that a variety of tasks are associated with telerehabilitation. Depending on the program, tasks before, during, and after a therapy session differ (e.g. technology installation, program adaptation or monitoring). It also varies whether these are performed by health professionals, patients, or technology. We identified, that users need digital, health-related, social, personal, and professional competencies. For successful applications, users need telerehabilitation, as well as technical and health-related knowledge. Health professionals need clinical expertise and practical experience in their job. Technical and communication skills are required, as well as self-management and reading/writing skills on the part of patients, and creativity and clinical skills (e.g. in coaching) on the part of health professionals. In addition to technology affinity and acceptance, motivation and commitment are conducive attitudes. A relevant personality trait is the confidence in one's own abilities. Whether sociodemographic factors such as age or gender promote successful technology use is discussed heterogeneously in the literature.

### Research gaps derived

Building on these findings, we identified research gaps. First, we only found a few articles reporting on health-related competencies. Regarding health literacy, only the required health knowledge is mentioned.^139^ This is insufficient since health literacy includes diverse skills that enable a profitable, thoughtful use of health services.^142,143^ Second, we found that health professionals need a range of social skills to remotely build rapport with patients and manage communication.^106^ What stands out is that the social skills of patients are not examined in detail. Third, we found that sociodemographic factors may impact successful usage. Interestingly, studies primarily analyse patients’ sociodemographic factors.^127,130^ Whether e.g. education or gender affects successful usage cannot be answered for health professionals.

### Exclusion and inclusion through telerehabilitation

Telerehabilitation offers access to rehabilitation for patients unable or unwilling to attend centre-based therapy,^144^ and thus can overcome access barriers such as mobility limitations.^145,146^ What remains unclear is whether telerehabilitation actually creates a more inclusive and equitable access/usage or whether existing inequalities in health care are being reinforced. In favour of more equitable access is the fact that programs are partly developed in a participatory design, and thus already involve patients and their competencies in the development process.^84,135^ Other programs are designed for people of all levels of competence, which speaks for a non-discriminating use of telerehabilitation.^33^ What speaks against it is the fact that only a minority of programs is developed participatory and thus do not take into account the diversity of patients.^112^ Further, we showed that a broad range of competencies is required for successful usage. Users who lack these have bigger challenges benefiting from telerehabilitation. At the same time, certain user groups are excluded from telerehabilitation. This affects people with no access to adequate hardware/equipment or with severe cognitive diseases.^57,113,140^ Our results are consistent with other studies indicating that people with a lower socioeconomic status, disabilities, limited health/digital literacy, in rural areas, or older people are more likely to lack access to telehealth or to not benefit from it.^147–150^

### Orientation towards user competencies

Telerehabilitation programs must consider users’ competencies. The included articles show that orientation towards competencies takes place before or during program usage. First, a needs-oriented technology development according to users’ competencies is necessary.^92^ Next, the selection of patients for telerehabilitation should take into account their competencies. Thus, it has to be decided whether a patient is more suited for analogue, hybrid, or digital services.^51,81,123^ Partly, health professionals and patients have the option to choose between different programs, technologies, or devices.^47,91^ In this case, the users’ competencies must be considered. During technology usage orientation towards users’ competencies can take place through adapting the program (e.g. therapy content, difficulty) to the patient.^51,86^

### Comparison with prior work

***eHealth literacy model.*** When comparing our results with the eHealth Literacy Model by Norman and Skinner,^19^ similarities and differences stand out. We found, that for telerehabilitation the following eHealth Literacies are relevant: traditional, computer, and health literacy (in particular self-management of health).^46,97,109^ The information, media, and science literacy were not addressed in the underlying studies of our review. This could be since these competencies focus on the selection of suitable media, research findings or information from a pool of available options, as well as their critical evaluation and usage.^19^ Patients at a rehabilitation facility often only have access to one program, so these selection/evaluation processes are not required for telerehabilitation.

***Other competence frameworks.*** As stated before, various competence frameworks exist in the field of telehealth. For example, Galpin et al.^14^ explore the telehealth skills of healthcare professionals in the United States. Many similarities with our results can be seen, regarding e.g. skills in remote clinical assessment, monitoring, treatment, and communication, as well as building relationships or demonstrating a good ‘web-side manner’. However, the competencies we have developed are more specific in that they distinguish between required knowledge, skills, personality traits, and attitudes. Hilty et al.^15^ present a telehealth competence framework for American behavioural health professionals. In contrast to our review, they distinguish between three levels of competence: novice, proficient, and expert. Accordingly, some of the required competencies we identified can be assigned to the expert level, such as ‘express empathy’ or ‘engage the patient’. Further, van Houwelingen et al.^16^ identified competencies for telehealth nurses in the Netherlands. Like us, they distinguish knowledge, attitudes, and skills. The competencies overlap strongly with those we identified, but they focus more broadly on competencies in seeking and using health information. Lastly, Helms et al.^17^ analyse the required competencies of telemedicine assistants in Germany. Competencies that differ from ours are resilience, abstraction ability, and professional distance-keeping.

### Future implications

***Implications for research.*** First, some open questions arise which require a more in-depth analysis in the future. While some identified competencies are particularly important in synchronous therapy (e.g. communication skills for videoconferencing^37^), a large proportion of articles was unspecific and related to telerehabilitation in general.^98,118^ Future research should examine how competencies differ by type of telerehabilitation. Further, the literature did not distinguish between essential or non-essential competencies, nor was the level of competence analysed. This should be specified in further surveys. As we have made clear, some competencies are discussed heterogeneously, such as sociodemographic factors.^67,78,127^ Delphi surveys could be used to build consensus among relevant stakeholders.

As mentioned above, a review is only the first step in developing a competence framework by generating a preliminary list of competencies.^10^ The next step is to involve the target groups and confirm the identified competencies. The development of a competence model for patients would be a great innovation, in particular because prior competence frameworks for telehealth have exclusively focused on health professionals so far.^14–17^ As we identified that competencies differ by indication and health profession, it could be useful to create competence profiles for different professions or different disease patterns/severities in the future. These could be used in the recruitment process of appropriate staff or the selection of patients for telerehabilitation.

***Implications for practice and policy.*** Regarding program development, the target groups and their competencies should always be involved by program producers (e.g. in user-centred participatory designs) to ensure successful use.^135^

Further, equitable access to and utilisation of high-quality rehabilitative services for all patients must be expanded. The use of rehabilitative technologies should therefore not exclude or disadvantage specific groups. From a political perspective, this also means expanding the technology infrastructure (especially in rural areas). Telerehabilitation should be an extension and no replacement for analogue services. In this sense, patients should be able to decide together with their health professionals and based on personal preferences and competencies, which model of rehabilitation (digital, in-person, or hybrid) they want to use.^34,147^

Subsequently, preparation, usage, and follow-up of telerehabilitation by patients should be supported to ensure success. This can include on-site, telephone or digital support, or the possibility of borrowing equipment. Patients and health professionals should be provided with education and training prior and during program usage.^149^ Regarding health professionals, telerehabilitation competencies should already be taught in vocational training.^57,115^

## Limitations

The article includes a few limitations. First, we did not evaluate the quality of the included studies and included less evidence-based content like discussion papers. Thus, the results are partly based on individual opinions, are not reliable regarding the causality or significance of effects and do not reflect a consensus among experts. Second, the analysis was complicated by the fact that relevant terms, like literacy, skills, abilities, or competencies, were partly used synonymously or not clearly distinguished from one another in the underlying articles. Third, the generalisability of results to specific jobs in specific countries is limited. This is due to different structures of (tele)rehabilitation systems in the countries. For example, different countries have different professions/jobs for the execution of teletherapy. Lastly, in deviation from the research protocol, we did not screen the reference list of identified articles due to time constraints. This could have resulted in not finding all relevant articles.

## Conclusions

We found that a variety of competencies – namely knowledge, skills, attitudes, and personal traits – are required to successfully use telerehabilitation. Competence frameworks do not yet exist in the field of telerehabilitation. The required competencies differ depending on the program and which tasks are performed by health professionals, patients, or the technology. Research gaps exist regarding the relevance of socio-demographic factors or different health-related competencies for both user groups and social competencies on the part of patients.

## Supplemental Material

sj-docx-1-dhj-10.1177_20552076231218841 - Supplemental material for Competencies required by patients and health professionals regarding telerehabilitation: A scoping review Click here for additional data file. Supplemental material, sj-docx-1-dhj-10.1177_20552076231218841 for Competencies required by patients and health professionals regarding telerehabilitation: A scoping review by Anna Lea Stark, Stephan Krayter and Christoph Dockweiler in DIGITAL HEALTH

sj-docx-2-dhj-10.1177_20552076231218841 - Supplemental material for Competencies required by patients and health professionals regarding telerehabilitation: A scoping review Click here for additional data file. Supplemental material, sj-docx-2-dhj-10.1177_20552076231218841 for Competencies required by patients and health professionals regarding telerehabilitation: A scoping review by Anna Lea Stark, Stephan Krayter and Christoph Dockweiler in DIGITAL HEALTH

sj-xlsx-3-dhj-10.1177_20552076231218841 - Supplemental material for Competencies required by patients and health professionals regarding telerehabilitation: A scoping review Click here for additional data file. Supplemental material, sj-xlsx-3-dhj-10.1177_20552076231218841 for Competencies required by patients and health professionals regarding telerehabilitation: A scoping review by Anna Lea Stark, Stephan Krayter and Christoph Dockweiler in DIGITAL HEALTH
