# Supplementary material for: Competencies required by patients and health professionals regarding telerehabilitation: A scoping review
Source: Digit Health. 2023 Dec 11;9:20552076231218841. doi: 10.1177/20552076231218841 (PMC10722929; doi:10.1177/20552076231218841)
Supplement: sj-docx-2-dhj-10.1177_20552076231218841 - Supplemental material for Competencies required by patients and health professionals regarding telerehabilitation: A scoping review [file sj-docx-2-dhj-10.1177_20552076231218841.docx]

**Appendix 2: Search Strategy MEDLINE (Stark et al. - Competencies required by patients and health professionals regarding telerehabilitation: a scoping review)**

(rehab*[Title/Abstract] OR telerehab*[Title/Abstract] OR postrehab*[Title/Abstract] OR e-rehab*[Title/Abstract] OR erehab*[Title/Abstract] OR tele-rehab*[Title/Abstract] OR post-rehab*[Title/Abstract] OR telerehabilitation[Mesh:NoExp]) AND (digital*[Title/Abstract] OR digitiz*[Title/Abstract] OR tele*[Title/Abstract] OR web-based[Title/Abstract] OR webbased[Title/Abstract] OR remote*[Title/Abstract] OR virtual*[Title/Abstract] OR internet*[Title/Abstract] OR mobile*[Title/Abstract] OR online*[Title/Abstract]) AND (competenc*[Title/Abstract] OR skill*[Title/Abstract] OR literac*[Title/Abstract] OR educat*[Title/Abstract] OR knowledge*[Title/Abstract] OR qualifi*[Title/Abstract] OR train*[Title/Abstract] OR abilit*[Title/Abstract] OR attitud*[Title/Abstract] OR value*[Title/Abstract])

Filter: 2017-2022
